# iFLinkC: an iterative functional linker cloning strategy for the combinatorial assembly and recombination of linker peptides with functional domains

**DOI:** 10.1093/nar/gkz1210

**Published:** 2020-01-11

**Authors:** Alexander Gräwe, Jan Ranglack, Anastasia Weyrich, Viktor Stein

**Affiliations:** 1 Fachbereich Biologie, Technische Universität Darmstadt, 64287 Darmstadt, Germany; 2 Centre for Synthetic Biology, Technische Universität Darmstadt, 64283 Darmstadt, Germany

## Abstract

Recent years have witnessed increasing efforts to engineer artificial biological functions through recombination of modular-organized toolboxes of protein scaffolds and parts. A critical, yet frequently neglected aspect concerns the identity of peptide linkers or spacers connecting individual domains which remain poorly understood and challenging to assemble. Addressing these limitations, iFlinkC comprises a highly scalable DNA assembly process that facilitates the combinatorial recombination of functional domains with linkers of varying length and flexibility, thereby overcoming challenges with high GC-content and the repeat nature of linker elements. The capacity of iFLinkC is demonstrated in the construction of synthetic protease switches featuring PDZ-FN3-based affinity clamps and single-chain FKBP12-FRB receptors as allosteric inputs. Library screening experiments demonstrate that linker space is highly plastic as the induction of allosterically regulated protease switches can vary from >150-fold switch-ON to >13-fold switch-OFF solely depending on the identity of the connecting linkers and relative orientation of functional domains. In addition, Pro-rich linkers yield the most potent switches contradicting the conventional use of flexible Gly-Ser linkers. Given the ease and efficiency how functional domains can be readily recombined with any type of linker, iFLinkC is anticipated to be widely applicable to the assembly of any type of fusion protein.

## INTRODUCTION

Synthetic biology aims to devise systematic approaches to engineer artificial biological functions for diverse biotechnological and biomedical applications. These range from tailored metabolic pathways that convert readily available carbon sources into higher value chemicals (1,2) to artificial signal transduction circuits capable of executing complex response functions (3,4). Yet, our understanding how a ‘genetic program’ at the level of DNA translates into tailored biological functions is still incomplete. The construction of biological functions thus still heavily relies on empirical optimization through iterative design-build-test and learn cycles. A key step concerns how ‘genetic programs’ are effectively ‘written’ either through the *de novo* synthesis of DNA or recombination of natural DNA fragments or a combination thereof. To this end, a growing number of DNA assembly methods can be applied to assemble synthetic or natural DNA fragments into large genetic circuits that are composed of multiple independent transcriptional units several kb in size (5).

DNA assembly methods can be broadly subdivided into homology-dependent (6–8) and restriction-enzyme based methods (9–11). The former relies on homologous dsDNA ends guiding the recombination of complementary strands of dsDNA; fusion of dsDNA can either be achieved naturally through endogenous DNA repair mechanisms—notably, in *Saccharomyces cerevisiae* (12), but also other microorganisms such as *Escherichiacoli* (13,14)—or in reconstituted form with key components of the DNA repair machinery purified (7,8) or preserved in bacterial cell extracts (6). Alternatively, short ssDNA ends generated by restriction enzymes can direct the ligation of any two DNA fragments. In particular, type IIS restriction enzymes that cut outside their recognition site form the basis of several seamless assembly methods such as Golden Gate (9) and derivatives thereof (10,11). Combined, these methods have been successfully applied to assemble large genetic circuits several kb in size that are composed of multiple transcription units (15–17).

In contrast, there is a distinct lack of toolboxes and DNA assembly methods tailored to the needs of proteins—arguably, because proteins display less functional modularity compared to transcription units that are composed of spatially distinct promoters, ribosomal binding sites, open reading frames and terminators. This view is rapidly changing as substantial efforts are made to develop modular-organized toolboxes of protein scaffolds and protein parts that enable the construction of complex protein functions (3,18). Such toolboxes comprise repositories and methods that facilitate the introduction of affinity tags and chaperones to assist with solubility, folding and purification of recombinant proteins (19), the assembly of metabolic enzymes and co-factors into chemical reaction cascades (20,21), and the compilation of synthetic protein switches into artificial signaling circuits that mediate biomolecular signals through the concerted action of a receptor coupled to an actuator (22–25). A key, yet frequently neglected factor in the assembly of the underlying fusion proteins concerns the identity of linkers connecting individual domains. Linkers have been shown to affect both structural and functional properties such as folding (26), proteolytic stability (27), flexibility (28,29) and relative 3D orientation of individual domains (30). Despite their central role in recombinant protein technology, there is a distinct lack of toolboxes and DNA assembly strategies that enable the scalable, combinatorial and high-fidelity recombination of functional domains through generic linker elements.

Addressing these limitations, a new DNA assembly process was devised enabling the systematic recombination of functional domains with arbitrary linker elements. The DNA assembly process—termed iterative functional linker cloning (iFlinkC)—relies on the combined action of type IIS restriction enzymes and T4 DNA ligase in order to fuse a functional domain with a linker separated by a single Gly residue in frame before regenerating the entry plasmid and thus enabling the iterative and combinatorial assembly of fusion proteins. The potential of iFlinkC is demonstrated in the construction of synthetic protease switches demonstrating a large plasticity underlying functional linker space. Library screening experiments also highlight the importance of Pro-rich linkers for optimal switching behaviour which contradicts the conventional use of flexible Gly-Ser linkers.

## MATERIALS AND METHODS

### General

The DNA coding for functional domains and linker elements was commercially synthesized as ssDNA or gBlocks (IDT DNA Technologies). The DNA coding for functional domains and linker elements was devoid of BsrDI, BtsI, EcoRI and SpeI restriction sites and cloned into pL2 and pFD vectors either by means of conventional restriction digestion ligation, Gibson assembly (8) or ligase cycling reaction (31). The function of tobacco vein mottling virus (TVMV) protease was assayed using a previously established assay based on the quenched fluorescent peptide substrate ANA-GETVRFQSDT(164)-NH_2_ (32–34). Here, ANA represents the fluorescence quenching 5-amino-2-nitrobenzoyl group and (164) the fluorescent 7-methoxycoumarinyl-4-acetyl group. The PDZ ligand NH_2_-RGSIDTWV-COOH (35) and the autoinhibitory domain NH_2_-EYVRFAP-NH_2_ and various derivatives with GPG linker extension—i.e. NH_2_-EYVRFAPG-NH_2_, NH_2_-EYVRFAPGP-NH_2_ and NH_2_-EYVRFAPGPG-NH_2_—were synthesized commercially (GenScript).

### iFLinkC assembly of linker libraries

All cloning enzymes were purchased from New England Biolabs (NEB). To generate combinatorial linker libraries, pL2 and pFD coding for different linker elements and functional domain were restriction digested with either BtsI or BsrDI and SpeI/EcoRI. The resultant DNA fragments were purified by agarose gel electrophoresis (Macherey-Nagel) and subsequently fused with T4 DNA ligase to regenerate the entry plasmid. To enhance the specificity of ligation, one set of DNA fragments was generally dephosphorylated using recombinant shrimp alkaline phosphatase. A list of linker elements and functional domains available for assembling combinatorial linker libraries by means of iFLinkC is summarized in Tables 1 and 2. Available elements comprise linkers of different lengths and flexibilities: e.g. flexible poly-Gly, intermediate poly-PAS (36) and rigid poly-Pro (37) and poly-TP (27,38). Detailed steps on the assembly of combinatorial linker libraries of individual synthetic protease switches are provided in the Supplementary Information (Supplementary Tables S1 and 2). The efficiency of ligation and subsequent transformation was generally monitored and sufficiently large to saturate library diversity (Supplementary Table S3).

**Table 1.** Summary of linker elements available with iFLinkC

Linker ID^a^	Amino Acid Sequence	Length	Description
	**Short**		
G	G	1	Short linkers are operationally defined to comprise three amino
G2	GG	2	acids or less (two of which constitute bridging Gly residues).
GPG	GPG	3	
G3	GGG	3	
	**Flexible**		
GGS4	GGSG	4	Flexible linkers are enriched in Gly and Ser residues of varying
GGS7	GGSGGSG	7	length.
GGS10	GGSGGSGGSG	10	
GGS15	GGSGGSGGSGGSGSG	15	
	**Rigid**		
P5	GPPPG	5	Rigid linkers feature poly-Pro, poly-PT and poly-Ala motifs
P9	GPPPPPPPG	9	stabilized by E/K salt-bridges.
TP11	GTPTPTPTPTG	11	
EAAAK16	GGAEAAAKEAAAKAGG	16	
	**Semi-Flexible**		
PAS5	GSPAG	5	Semi-flexible linkers featuring a PASylation-motif
PAS8	GGASPAGG	8	(a combination of Pro, Ala and Ser).
PAS12	GGASPAAPAPAG	12	

^a^Linker IDs are comprised of 2–5 letter code capturing the most characteristic amino acid or amino acid repeat followed by the length of the linker (including the two bridging Gly residues). Short linkers with fewer than three amino acids and no obvious repeats are generally spelt out.

**Table 2.** Summary of functional domains available with iFLinkC

Name	Length
FRB	91
FN3	92
ePDZ-B1	97
FKBP12	106
MBP-CS^TEV^-StrepTag-II-AI^TVMV^	398
TVMV Protease	222

### Screening protease switches

Libraries of protease switches were transformed into electrocompetent BL21 (DE3) and plated on LB agar plates supplemented with 100 μg/ml ampicillin. Following overnight incubation at 37°C, single colonies were inoculated into 96 deep-well plates filled with 650 μl modified minimal autoinduction N-5052 medium (0.5% glycerol, 0.05% glucose, 0.2% lactose, 50 mM KH_2_PO_4_, 50 mM Na_2_HPO_4_, 10 mM (NH_4_)_2_SO_4_, 1 mM MgSO_4_ and 1 × trace metal solution) supplemented with 100 μg/ml ampicillin (39). Proteins were left to express for 72 h at 37°C in a multi-well plate shaker at 1.200 rpm. Following expression, 100 μl of each culture was saved as glycerol stocks while the remaining cells were spun down and the resultant pellets stored at −20°C. Cell lysates of *E. coli* for screening were prepared as previously published (40): Briefly, pellets were resuspended in 100 μl Buffer W (150 mM NaCl, 100 mM Tris–HCl, 1 mM ethylenediaminetetraacetic acid (EDTA) pH 8.0) supplemented with 1 mg/ml lysozyme and 1 μg/ml DNase, and then incubated for 2 h at 30°C in a multi-well plate shaker at 1.200 rpm. The lysate was cleared of cell debris by centrifugation for 20 min at 2.500 × *g* and 4°C before assaying the function of protease switches in 96-well plate: Briefly, 20 μl lysate supernatant was added to 90 μl Buffer A (150 mM NaCl, 100 mM Tris–HCl, 4 mM DTT and 5 mM EDTA pH 8.0) and pre-incubated for 5 min at room temperature in the presence and absence of 5 μM rapamycin or 10 μM PDZ ligand. The assay was initiated through the addition of 90 μl Buffer A supplemented with 5 μM TVMV peptide substrate. Cleavage of the TVMV peptide substrate was monitored by means of fluorescent spectroscopy in a 96-well plate reader (TECAN Spark) at 30°C and *λ*_Ex/Em_ at 330 nm and 405 nm.

### Protein expression and purification

For detailed characterization, selected mutants identified in library screening experiments were expressed in BL21(DE3) in 1 L LB medium supplemented with 100 μg/ml ampicillin. Protein expression was induced with 1 mM IPTG when cells reached an OD_600_ of 0.5 and left to express for 4.5 h at 30°C and 220 rpm. Cultures were harvested by centrifugation at 2.500 × *g* at 4°C and stored at −20°C. For purification, cells were resuspended in 40 ml Buffer W (150 mM NaCl, 100 mM Tris–HCl, 1 mM EDTA, pH 8.0) and crushed via four passes through an Emulsiflex C3 (Avestin). After centrifugation at 25 000 × *g* for 1 h at 4°C, the resultant lysates were filtered through a 0.45 μm nitrocellulose filter. Protease switches were purified by means of affinity chromatography using an automated AKTA pure L chromatography system on 1 ml StrepTrap HP column according to manufacturer's instructions (GE Healthcare). The protein was eluted in Buffer E (150 mM NaCl, 100 mM Tris–HCl, 2.5 mM desthiobiotin, 1 mM EDTA, pH 8.0). Protein aliquots were flash frozen in liquid nitrogen and stored at −80°C until further use.

### Functional characterization of synthetic protease switches

The activity of allosteric TVMV switches was assayed in Buffer A (150 mM NaCl, 100 mM Tris–HCl, 4 mM DTT and 5 mM EDTA pH 8.0) supplemented with 5 μM TVMV substrate in a total volume of 200 μl. Cleavage of the TVMV substrate was monitored by means fluorescent spectroscopy in a 96-well plate reader (TECAN Spark) at 30°C and *λ*_Ex/Em_ at 330 and 405 nm. The concentration of individual TVMV switches is indicated for individual experiments. For long measurements with 20 nM TVMV or less, the assay was additionally supplemented with 50 μg/ml bovine serum albumin to prevent non-specific adsorption. Maximum induction ratios were measured as the fold-ratio in the presence and absence of saturating ligand concentrations (either 5 μM rapamycin or 10 μM PDZ ligand) when the fluorescence signal displayed a linear range above background. To quantify apparent *K*_D_s, the activity of allosteric TVMV switches was measured across a concentration of 50 nM to 1 mM rapamycin. The *K*_D_s were determined by non-linear regression fit of the initial rates (Equation 1, Supplementary Data). To quantify the *K*_i_s of chemically synthesized AI-domains, the activity of TVMV was measured across a concentration of 50 nM to 1 mM AI-based inhibitors as indicated. *K*_i_ were determined by non-linear regression fit of the initial rates (Equation 2, Supplementary Data).

## RESULTS

### iterative Functional Linker Cloning (iFLinkC)

**Figure 1.**
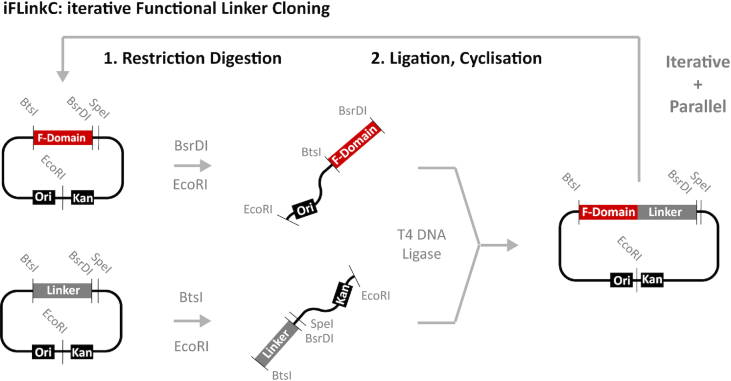
Overview of an elementary iFlinkC DNA assembly cycle: A functional domain is fused with a linker element through the combined action of BtsI, BsrDI and either EcoRI or SpeI before regenerating the original plasmid through ligation, which enables the iterative, parallel and combinatorial assembly of fusion proteins and fusion protein libraries.

A new DNA assembly process along with a molecular toolbox was developed to facilitate the systematic assembly of fusion proteins and thereof derived linker libraries (Figure 1). Protein domains and linkers are first cloned into functionally equivalent entry plasmids termed pL2 and pFD. Sequences coding for linkers and functional domains are flanked by type IIS restriction enzymes BtsI and BsrDI that cleave adjacent to their cognate DNA sequence. Crucially, BtsI and BsrDI both generate a two base pair overhangs and thus allow in frame fusion of any two proteins through a minimal Gly residue. Fusion proteins are then assembled iteratively following a rule-based protocol using plasmid DNA stored in locally, sequence verified repositories independent of a polymerase chain reaction (PCR) step and the purchase of synthetic DNA: Briefly, pFD and pL2 are each digested with either BtsI or BsrDI and SpeI/EcoRI depending on the size of the insert and subsequently gel purified. The DNA coding for the functional domain and the linker are subsequently ligated in frame to regenerate the entry plasmid. Crucially, by leaving comparatively short linker elements fused to a larger DNA fragment, iFlinkC enables the simultaneous fusion of very short and very long linkers. In addition, since the original entry backbone is regenerated following ligation, the process can be repeated iteratively and in parallel to rapidly generate combinatorial fusion protein libraries. At last, the fully assembled fusion protein libraries are inserted into a destination vector, pFLinkC-XE, to express the protein of interest in an organism of choice. A list of linker elements and functional domains available for combinatorial assembly with iFLinkC is summarized in Tables 1 and 2.

### Engineering modular-organized protease switches with iFLinkC

**Figure 2.**
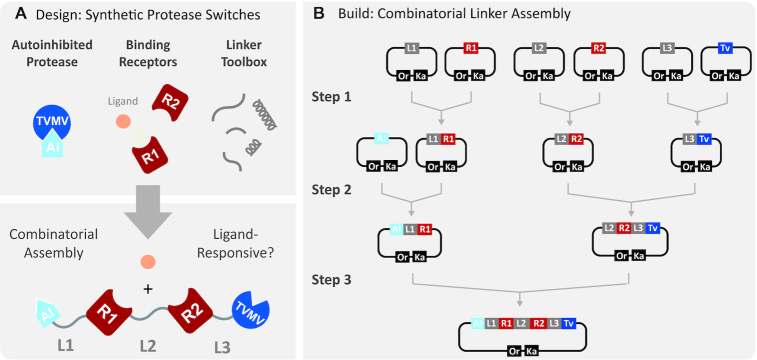
(**A**) Synthetic protease switches with tailored response functions are engineered through recombination of autoinhibited protease modules with structurally distinct binding receptors and peptide linkers of varying flexibility and length; (**B**) Combinatorial 3-step assembly of a synthetic protease switch by means of iFLinkC: Functional domains coding for autoinhibited protease modules, binding receptors and defined linker elements are stored in sequence verified entry vectors. Individual components are fused in-frame in pairwise assembly reactions. Since the original entry plasmid is regenerated following every assembly cycle and the number of different elements included in any pairwise assembly reaction is not limited, iFLinkC enables the combinatorial assembly of modular organized protease switches, and more generally multi-domain fusion proteins as well.

With a reliable working protocol established, iFLinkC was applied to construct a set of synthetic protease switches based on a recently developed toolbox of autoinhibited protease modules (32–34). The system is highly modular as proteases along with their cognate autoinhibitory (AI) domains can be recombined with structurally distinct receptors to engineer sensors and switches with tailored inputs (Figure 2A). A key question concerns how the relative orientation and the identity of linker elements connecting functional domains shapes the response function of protease switches. In this regard, current efforts are however hampered by a lack of suitable assembly methods to generate combinatorial linker diversity at the level of DNA. Filling this technological gap, iFLinkC enables the assembly of defined combinatorial libraries through the iterative fusion of a functional domain (e.g. a receptor, a protease or its cognate AI-domain) with distinct linkers (Figure 2B). A combinatorial linker library composed of four functional domains connected by three linker elements can be readily realized within a 3-step-assembly process, and in theory expanded toward more complex fusion proteins independent of the relative orientation of functional domains and linker elements.

### Engineering PDZ-FN3 affinity clamp protease receptors

To validate iFLinkC in practice, a set of allosterically regulated proteases was constructed featuring PDZ-FN3 affinity clamps as allosteric input modules. Affinity clamps comprise a versatile class of allosteric receptors composed of a circular permutated PDZ domain, termed ePDZ-b and an enhancer domain, FN3, that form a clamp shell complex around their cognate peptide ligand (35,41). In a previous study, limited linker truncation yielded allosterically regulated TVMV proteases that could be induced between 5-fold switch-OFF and 30-fold switch-ON, yet were limited by high background activity in cell lysate assays. Background activity was presumably caused due to constitutively active TVMV lacking an AI-domain as a result of incomplete translation or non-specific proteolysis (32).

**Figure 3.**
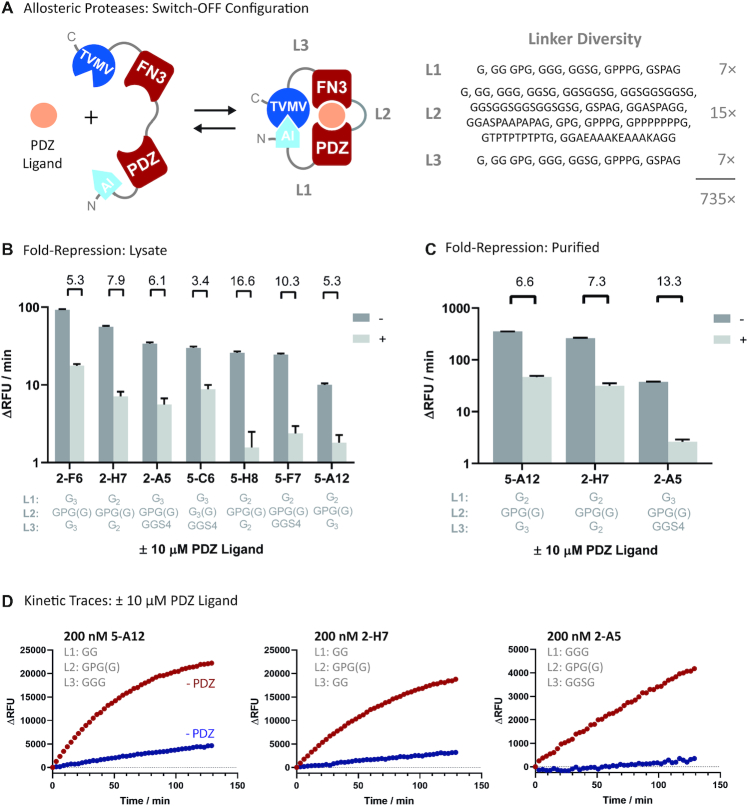
Construction of allosterically regulated protease switches regulated through PDZ-FN3 affinity clamps: (**A**) Screening 467 yielded a limited number of protease switches that were repressed upon addition of the cognate PDZ ligand (switch-OFF configuration); (**B**) Summary fold-repression (log scale) of seven different mutants assayed in cell lysates. Standard errors derive from linear regression analysis; (**C**) Summary fold-repression (log scale) of three different protease switches 2-A5, 5-A12 and 2-H7 assayed in purified form at 200 nM each. Standard errors derive from duplicate measurements. (**D**) Representative kinetic traces of three different protease switches 2-A5, 2-A12 and 2-H7 assayed in purified form at 200 nM each; Activities of allosteric protease switches are displayed and quantified in relative fluorescence units (RFUs) associated with the cleavage of the quenched fluorescent TVMV substrate.

To examine how peptide linkers shape the response function of PDZ-FN3-regulated protease switches, a combinatorial library featuring seven short linkers in L1 and L3, and 15 linkers of variable flexibility and length in L2 was assembled by means of iFLinkC (Figure 3A and Supplementary Table S1). To limit non-specific background activity, the position of the AI-domain was also relocated from the C- to the N-terminus. A sub-saturating number of 467 variants (out of a theoretical diversity of 735) was subsequently screened in multi-well plates in the presence and absence of the PDZ ligand NH_2_-RGSIDTWV-COOH. The library screen yielded TVMV switches that were primarily repressed upon addition of the cognate PDZ ligand (Figure 3A). Six variants that were repressed >5-fold were sequenced and converged on a non-conventional GPG(G)-motif in L2 separating the PDZ and the FN3 domain while L1 and L3 generally featured short linkers (Table 3, Figure 3B and Supplementary Figure S1). One further switch featured a GGG(G)-linker and could be repressed ∼3-fold (Figure 3B and Supplementary Figure S2). Three variants identified in the cell lysate assay AI^G2^-PDZ^GPG-G^FN3^G3^-TVMV (5-A12), AI^G3^-PDZ^GPG-G^FN3^GGS4^-TVMV (2-A5) and AI^G2^-PDZ^GPG-G^FN3^G2^-TVMV (2-H7) were purified and characterized further: Variant AI^G3^-PDZ^GPG-G^FN3^GGS4^-TVMV (2-A5) showed strong >13-fold repression upon addition of the PDZ ligand while AI^G2^-PDZ^GPG-G^FN3^G3^-TVMV (5-A12) and AI^G2^-PDZ^GPG-G^FN3^G2^-TVMV (2-H7) could be repressed ∼7.6- and 8.3-fold, respectively (Figure 3C, Figure 3D and Table 1). Notably, the four amino acid GPG(G)-motif deviates from previously identified OFF-switches that were largely insensitive to the identity of the linkers connecting the PDZ with the FN3 domain (32). Overall, this highlights a comparatively large, yet idiosyncratic plasticity underlying the response function of modular organized protease switches both in response to the composition of linkers and the relative orientation of functional domains.

**Table 3.** Outcomes: library screening experiments AI^L1^-PDZ^L2^-FN3^L3^-TVMV

Clone ID	L1	L2	L3	×-Fold Repression (Lysates)	×-Fold Repression (Purified)
5-H8	GG	GPG(G)	GG	16.6 ± 9.9	
5-F7	GG	GPG(G)	GGSG	10.3 ± 2.5	
2-H7	GG	GPG(G)	GG	7.9 ± 1.2	8.3 ± 1.0
2-A5	GGG	GPG(G)	GGSG	6.1 ± 1.3	14.3 ± 1.5
5-A12	GG	GPG(G)	GGG	5.5 ± 1.2	7.6 ± 0.4
2-F6	GGG	GPG(G)	GGG	5.2 ± 0.3	
5-C6	GGG	GGG(G)	GGSG	3.4 ± 0.5	

### Engineering FKBP12-FRB protease receptors

The general utility of iFlinkC was further demonstrated in the construction of allosterically regulated proteases featuring rapamycin-responsive, single-chain FRB-FKBP12 receptors as input modules. The FKBP12-FRB protein–protein interaction module is widely used for co-localizing proteins in a rapamycin-dependent fashion (42,43). Recently, an allosteric, single-chain FKBP12-FRB receptor was developed to confer direct control over protein function independent of a technically challenging protein co-expression system (44,45). Here, computational design guided the recombination of FKBP12 with FRB before inserting the resultant receptor, termed uniRapR, into conserved catalytic loops of several mammalian protein kinases to control their activity with rapamycin (44,45). While the approach yielded a set of powerful tools to examine the mechanisms of protein kinase signaling in live cells, the general utility of the design strategy is limited as it relies on the exchange of secondary structural elements between the FKBP12 and FRB and the insertion of the resultant receptor, uniRapR, into the tertiary structure of protein kinases, the outcome of which is considered unpredictable on protein fold, structure and function.

**Figure 4.**
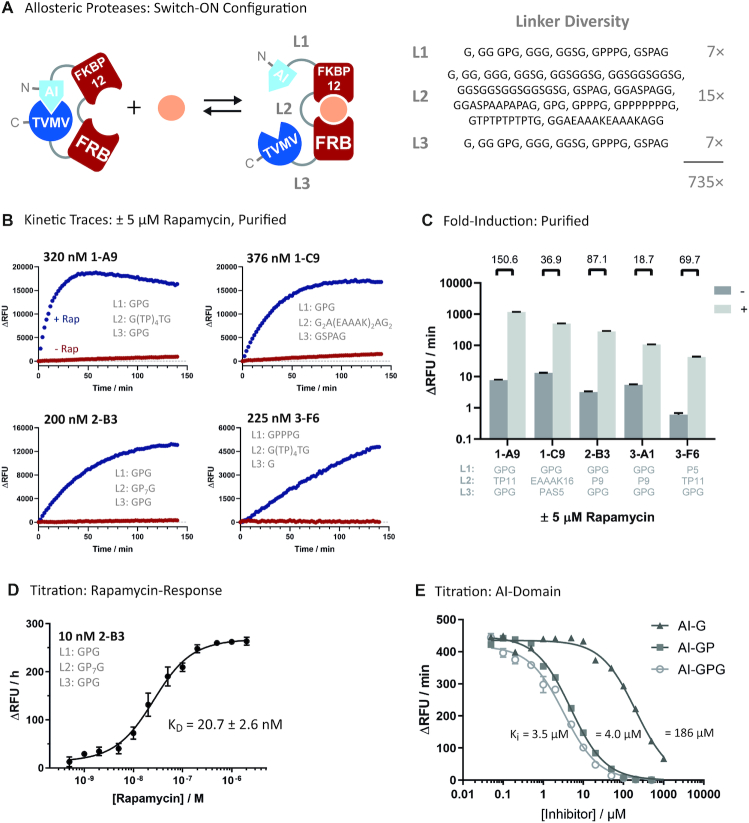
Construction of allosterically regulated protease switches regulated through single-chain FKBP12-FRB receptors. (**A**) Screening 230 variants yielded a limited number of protease switches that were induced upon addition of rapamycin (switch-ON configuration); (**B**) Representative kinetic traces of a select number of variants 2-B3, 1-A9, 3-C9 and 3-F6 assayed in purified form at the indicated concentrations; (**C**) Summary fold-induction (log scale) of five different variants 2-B3, 1-A9, 3-C9, 3-A1 and 3-F6 in purified form. Standard errors derive from duplicate measurements; (**D**) Titration of the rapamycin response for TVMV switch 2-B3. Standard errors derive from duplicate measurements at each rapamycin concentration; (**E**) Determining *K*_i_ of different derivatives of the AI-domain; Activities of allosteric protease switches are displayed and quantified in relative fluorescence units (RFUs) associated with the cleavage of the quenched fluorescent TVMV substrate.

Instead, a fully modular approach was pursued with the aim of engineering rapamycin-responsive TVMV switches based on structurally distinct FKBP12 and FRB domains while only varying the relative orientation of functional domains and composition of the connecting linkers. To this end, a combinatorial linker library featuring FKBP12 and FRB in an N- to C-terminal orientation was assembled by means of iFLinkC. To limit non-specific background activity from incompletely translated protein, the AI-domain and TVMV were placed at the N- and C-terminus, respectively. Combinatorial linker libraries were designed following a structure-guided hypothesis assuming that the largest movement occurs between FKBP12 and FRB, thus choosing short linkers in L1 and L3, and linkers of variable length and flexibility in L2 (Figure 4A and Supplementary Table S2).

Analogous to the library screening experiments with PDZ-FN3-based TVMV switches, a sub-saturating number of 230 AI-FKBP12-FRB-TVMV mutants was screened for maximum response following the addition of 5 μM rapamycin. The resultant protease switches predominantly displayed switch-ON behavior reaching induction ratios >100-fold in cell lysates while remaining strongly suppressed in the basal OFF-state in the absence of rapamycin (Table 4 and Figure 4B). To gain a better understanding of the molecular features that underlie switching function, 22 mutants with distinct switching behaviour were sequenced (Table 4 and Supplementary Figures S3–6). Strikingly, the best variants of the AI-FKBP12-FRB-TVMV library converged on a short GPG-motif in L1 giving rise to allosteric protease switches that were strongly suppressed in the basal state (Table 4 and Figure 4B). Short linkers were also observed in L3 while relatively long linkers featuring rigid poly-Pro, poly-TP and (EAAAK)_N_ helical motifs were enriched in L2. Notably, any switch that could be induced >12-fold featured no more than one flexible or semi-flexible linker (Table 4). Analysis by means of sodium dodecyl sulphate-polyacrylamide gelelectrophoresis demonstrated that protein yields were high under conditions of recombinant overexpression in *E. coli* including TVMV switches featuring poly-Pro repeats (Supplementary Figure S7). Overall, these findings contradict the conventional use of flexible, Gly-rich linkers and point towards rigid, hinge-like linkers as optimal solutions in the construction of protein switches. Grouping protease switches according to common L2 linkers also demonstrates that more flexible linkers in L1 and L3 result in less suppressed and therefore less inducible protease switches (Supplementary Table S4).

**Table 4.** Outcomes: library screening experiments AI^L1^-FKBP12^L2^-FRB^L3^-TVMV

Clone ID	L1	L2	L3	×-Fold Induction (Lysates)	×-Fold Induction (Purified)
1-A9	GPG	GTPTPTPTPTG	GPG	>80	150.6 ± 5.1
2-B3	GPG	GPPPPPPPG	GPG	72.5 ± 13.7	87.1 ± 6.0
3-F6	GPPPG	GTPTPTPTPTG	G	68.3 ± 17	69.7 ± 8.9
3-A1	GPPPG	GPPPG	GG	64.4 ± 21.0	18.7 ± 1.0
1-C9	GPG	GGAEAAAKEAAAKAGG	GSPAG	>60	36.9 ± 0.7
3-C9	GPG	GGG	GSPAG	53.9 ± 9.2	
2-E7	GPG	GPPPPPPPG	G	41.0 ± 6.6	
3-F3	GPG	GSPAG	GGG	38.5 ± 7.5	
3-H4	GPG	GGSGGSGGSGGSGSG	GPPPG	21.6 ± 3.4	
2-G2	GPPPG	GGAEAAAKEAAAKAGG	GG	16.8 ± 1.9	
2-E3	GSPAG	GGAEAAAKEAAAKAGG	GSPAG	11.9 ± 0.9	
1-A7	GPPPG	GG	GGSG	5.0 ± 0.4	
2-G1	GSPAG	GGAEAAAKEAAAKAGG	GPPPG	4.2 ± 0.5	
1-A8	GGSG	GPG	GGSG	4.1 ± 0.2	
1-D11	GPPPG	GG	GSPAG	3.6 ± 0.5	
1-C11	GPPPG	GGSGGSGGSGGSGSG	GSPAG	3.6 ± 0.3	
1-D7	GSPAG	GGASPAAPAPAG	GSPAG	3.2 ± 0.1	
1-D10	GPPPG	GGASPAAPAPAG	GSPAG	3.0 ± 0.2	
3-A3	GSPAG	GGSG	GPPPG	2.8 ± 0.4	
3-D6	GSPAG	GGG	GPPPG	2.6 ± 0.1	
1-D4	GPPPG	GPG	GSPAG	2.6 ± 0.2	
1-E8	GSPAG	GGSGGSGGSGGSGSG	G	2.4 ± 0.2	

Five TVMV switches were subsequently purified and their function assayed *in vitro* confirming some of the best performing allosterically regulated protein switches developed to date with induction ratios in the range of 16- to >150-fold (Figure 4C). In addition, one of the top candidates 2-B3 featuring an unconventional poly-Pro linker was characterized further determining its affinity for rapamycin in a clamp-shell autoinhibited protease complex with an apparent *K*_D_ of 20 ± 3 nM (Figure 4D). In addition, given the strong suppression observed for GPG-motif in L1, the strength of binding was quantified in titration experiments with chemically synthesized AI-domain derivatives with single amino acid extensions of the GPG-motif (Figure 4E). Indeed, elongating the AI-domain EYVRFAP with either GP or GPG decreased the *K*_i_ of the AI-domain for TVMV by more than one order of magnitude from 196 to 4.5 ± 0.3 μM and 3 ± 0.2 μM, respectively. In contrast, a single Gly extension had no significant impact on affinity with a *K*_i_ = 186 ± 15 μM compared to the original AI-domain of 196 μM. Overall, the enhanced AI-domain can now be used for the construction of strongly repressed TVMV switches.

## DISCUSSION

With an increasing emphasis on developing post-translationally regulated biological functions, current efforts focus on devising generally applicable approaches through recombination of modular, well characterized proteins. A key, yet frequently neglected aspect concerns the identity of linkers connecting individual domains: Notably, only few studies have systematically explored how linkers impact protein function to date. In addition, strategies for generating combinatorial linker libraries are limited as conventional approaches for introducing genetic diversity into proteins (46) such as saturation mutagenesis (47), error-prone PCR (48) and recent advancements such as Darwin Assembly (49) target chemical diversity at distinct amino acids located in binding interfaces, catalytic centers or randomly distributed throughout a protein. In contrast, linker function heavily depends on length while chemical diversity is limited to combinations of amino acid repeats that confer distinct biophysical properties, for instance, on the capacity of a protein to fold correctly or undergo conformational changes (26–30). The effect of linkers thus turns out to be highly context-dependent and difficult to predict. At the same time, methods to generate linker diversity have been limited to single linker elements varying their length through limited restriction digests (50), homologous recombination (29) or PCRs with staggered sets of forward and reverse primers (51). The chemical and biophysical diversity that can be generated with these approaches is however limited as the component linkers typically derive from a single, larger linker element of synthetic (29,50) or natural origin (51). Effective assembly cloning and sequencing of linkers is additionally hampered by the high GC-content and the high number of repeat elements. At last, methods to generate combinatorial linker diversity in a systematic fashion are generally missing from the linker assembly toolbox.

Addressing these limitations, iFLinkC comprises an iterative DNA assembly process for the rapid generation of combinatorial linker diversity based on well-defined linker elements. Iterative fusion of functional domains with arbitrary linker elements is achieved through the combined action of type IIS restriction enzymes and a T4 DNA ligase before regenerating the original entry plasmid. This enables iterative and parallel assembly of multi-domain fusion proteins with a number of key advantages: First, the use of type IIS restriction enzymes overcomes any technical challenges associated with cloning GC-rich linker repeats by means of homology-dependent cloning methods. Second, iFLinkC enables the simultaneous fusion of very short and very long linkers. Third, functional domains and linkers are stored in sequence verified repositories reducing time and costs associated with the design and purchase of synthetic DNA as generic linker elements can be readily reused. Building repositories of defined linker elements also confers greater control over the chemical and biophysical properties that can be applied in the recombination of functional domains and thus applied in any linker engineering effort. Fourth, linker elements fused to functional domains can be stored in intermediate libraries and readily re-used in subsequent construction efforts. Fifth, iFLinkC can be readily combined with other library assembly methods. For instance, the bridging Gly residues may—by virtue of contributing to the linker—have a greater effect on the biophysical properties of shorter linkers. If this was a concern, or turned out limiting, any two fragments could still be fused by alternative library assembly methods (e.g. homology-dependent methods based on synthetic DNA featuring fully- or partially randomized codons in a linker). Such sub-libraries can be readily generated in iFLinkC entry vectors and easily integrated into the iFLinkC assembly process.

The capacity of iFLinkC is demonstrated in the construction of allosteric protease switches examining how their response varies as a function of linker composition and relative orientation of functional domains. To this end, affinity clamp and a modular version of a single-chain FKBP12-FRB receptor served as allosteric inputs. Library screening experiments yield some of the best performing allosteric protein switches developed to date with induction factors greater >100-fold and very low background activities in the basal state. Library screening experiments also highlight key design principles that underlie the most potent protein switches, in particular, the importance of the relative orientation of functional domains and the critical role of the component linkers. Notably, the best functional switches are biased toward Pro-rich linkers contradicting the conventional use of flexible Gly-rich linkers that – with a few exceptions such as LUCIDs with a Pro_30_ (52,53) and antibody-specific sensors with α-helical (EAAAK)_N_ linker elements (54–56)—have been preferred to date.

At last, iFlinkC also paves a general route for the step wise construction of synthetic protein switches with tailored inputs by, first, generating binders against an analyte of interest and, second, recombining binders with an actuator through combinatorial linkers. Crucially, library screening experiments demonstrate that highly potent switches occur sufficiently frequent so they can be readily identified through limited screening in multi-well plate formats. Given the ease and speed by which combinatorial linker diversity can be generated by means of iFLinkC, the devised methodology should be readily applicable to a number of different proteins and greatly facilitate foundational protein engineering studies how the identity of linkers impacts structural and functional properties of complex fusion proteins.

## DATA AVAILABILITY

Not applicable.

## Supplementary Material

gkz1210_Supplemental_File
