# Supplementary material for: iFLinkC: an iterative functional linker cloning strategy for the combinatorial assembly and recombination of linker peptides with functional domains
Source: Nucleic Acids Res. 2020 Jan 11;48(4):e24. doi: 10.1093/nar/gkz1210 (PMC7039005; doi:10.1093/nar/gkz1210)
Supplement: gkz1210_Supplemental_File [file gkz1210_supplemental_file.pdf]

Supplementary Information

iFLinkC: An iterative Functional Linker Cloning Strategy for the Combinatorial Assembly and Recombination of Linker Peptides with Functional Domains

Alexander Gräwe[1], Jan Ranglack[1], Anastasia Weyrich[1] and Viktor Stein[1*]

[1] Fachbereich Biologie, TU Darmstadt, 64287 Darmstadt, Germany

[*]Correspondence should be addressed to Viktor Stein:

Tel: +49 (0) 6151 16 21947; Fax: -; Email: stein@bio.tu-darmstadt.de

iFLinkC Assembly Cycle (Step-by-Step Protocol)

1. **General**

   Enzymes are generally purchased from New England Biolabs (NEB). To prepare and propagate plasmid DNA, *E.coli* was grown in Lysogeny Broth (LB) medium containing 1% tryptone (w/v), 0.5 yeast extract (w/v) and 1% NaCl (w/v). Agarose gel electrophoresis is performed in 1× TAE medium containing 40 mM Tris-Base, 20 mM AcOH and 1 mM EDTA. Recovery and outgrowth of *E.coli* following transformation is generally performed in super optimal broth (SOC) medium containing 2% tryptone (w/v), 0.5% yeast extract (w/v), 10 mM NaCl, 2.5 mM KCl, 10 mM $MgCl_2$, 20 mM $MgSO_4$ and 20 mM glucose.

2. **Restriction Digest DNA Fragments with BtsI and BsrDI**

   First of all, devise an assembly process deciding on the order of functional domains and linker elements in the fusion protein (see **Fig. 2** in the main manuscript). Functional domains are generally stored in pFD while linker elements are stored in pL2. Please note, pFD and pL2 are functionally equivalent enabling the direct fusion of any two functional domains or linkers.

   Once an assembly process has been devised, set up restriction digests using a combination of BtsI, BsrDI and either SpeI or EcoRI: The N-terminal DNA fragment is always digested with BsrDI while the C-terminal DNA fragment is always digested with BtsI. Depending on the size of the anticipated C-terminal DNA fragment either EcoRI or SpeI can be employed. For shorter DNA fragments, we recommend EcoRI while for longer DNA fragments >400 bp that are amenable to gel purification by themselves, we recommend SpeI.

   To prevent re-ligation of the entry plasmids in the subsequent ligation step, we recommend dephosphorylating one of the two restriction digests with recombinant shrimp alkaline phosphatase (rSAP). Instead of treatment with rSAP, plasmids may also be restriction digested with BbsI and/or BsaI to prevent re-ligation of the entry plasmid.

   Restriction digest with BsrDI (in a total volume of 50 µL; exemplified with pL2):

- Add equimolar amounts of purified pL2 coding for different linker elements. The total DNA mass should not exceed 1.2 µg.
- Fill up to a volume of 43 µL with MQ water.
- Add 5 µL 10× CutSmart buffer (NEB).
- Add 1 µL BsrDI (5 U) at 65 °C in NEB Buffer 2.1 for 1 h.
- Cool down to 37 °C and add 1 µL SpeI-HF (20 U/µL) or EcoRI-HF (20 U/µL).
- Add 1 µL rSAP (1 U/µL) and continue incubation for 60 min at 37 °C[1].

   Restriction digest with BtsI (in a total volume of 50 µL; exemplified with pFD):

- Add 1.2 µg purified pFD.
- Fill up to a volume of 43 µL with MQ water.
- Add 5 µL 10× CutSmart buffer (NEB).
- Add 1 µL BtsI (10 U/µL).
- Depending on assembly strategy, add 1 µL SpeI-HF (20 U/µL) or EcoRI-HF (20 U/µL).
- Incubate restriction digest for 90 min at 37 °C[2].

[1] Alternatively, the BtsI digest may be dephosphorylated.

[2] Although highest efficiency for BtsI is 55 °C, BtsI also works at 37 °C (NEB: 75%)

3. Purification of DNA Fragments by Agarose Gel Electrophoresis

- Stop restriction digest by adding 10 µL DNA loading dye (6x stock) to 50 µL restriction digest.
- Separate DNA fragments by means of agarose gel electrophoresis (for approx. 40 min at 120 V on 1% agarose in 1x TAE buffer). Depending on the size, DNA fragments may also be separated for a longer period of time and reduced voltage.
- Excise the desired DNA fragments using a clean scalpel.
- Extract DNA fragments from agarose gel, preferably with a commercial gel extraction kit such as NucleoSpin (Macherey-Nagel).

4. iFLinkC Assembly Reaction: Ligation of pFD and pL2 Fragments

- Add equimolar amounts of gel purified pFD and pL2. Total amount of DNA should be between 5 ng and 100 ng.
- Fill up to a volume of 10 µL with MQ.
- Add 1.2 µL T4 DNA Ligase buffer (NEB).
- Add 0.8 µL T4 DNA Ligase (NEB).
- Incubate ligation reaction at 16-18 °C either for 1 h or overnight.
- Heat-inactivate ligation reaction at 65 °C for 20 min.

5. Transforming iFLinkC Assembly Reaction

- Transform 2-3 µL of the iFLinkC assembly reaction into 50 µL aliquots of transformation-competent *E. coli*. Transformation can either be realised by means of heat-shock or electroporation.
- Perform outgrowth in 1 mL SOC medium for 1 h at 37 ˚C.
- To assess the efficiency of transformation, plate 40 µL on LB agar plate supplemented with 50 µg/mL kanamycin. The fidelity of the assembly reaction may also be confirmed by sequencing of individual clones.
- Use the remaining 960 µL to inoculate 10 mL LB supplemented with 50 µg/mL kanamycin and overnight incubation at 37 ˚C.

6. Plasmid preparation

- Harvest *E.coli* by centrifugation and purify plasmid library by means of commercial plasmid purification kit such as NucleoSpin (Machery-Nagel).

Table S1. Library Assembly of Affinity Clamp TVMV Switches: AI-PDZ-FN3-TVMV

Step	Ligation	N-Terminal Fragment(s)	C-Terminal Fragment(s)
1	1	--- BsrDI + **SpeI** + rSAP ---<br>Linker^{7S}<br>G, GG, GGG, GGSG, GPG, GPPPG, GSPAG	--- BtsI + **SpeI** ---<br>ePDZ-B1
	2	--- BsrDI + **SpeI** + rSAP ---<br>Link^{15L}<br>G, GG, GGG, GGSG, GGSGGSG, GGSGGSGGSG, GGSGGSGGSGGSGSG, GSPAG, GGASPAGG, GGASPAAPAPAG, GPG, GPPPG, GPPPPPPPG, GTPTPTPTPTG, GGAEAAAKEAAAKAGG	--- BtsI + **SpeI** ---<br>FN3
	3	--- BsrDI + **SpeI** + rSAP ---<br>Link^{7S}<br>G, GG, GGG, GGSG, GPG, GPPPG, GSPAG	--- BtsI + **SpeI** ---<br>TVMV
2	1	--- BsrDI + **EcoRI** + rSAP ---<br>MBP-CS^{TEV}-StrepTag-II-AI^{TVMV}	--- BtsI + **EcoRI** ---<br>Ligation Product 1.1<br>Linker^{7S}- ePDZ-B1
	2	--- BsrDI + **SpeI** + rSAP ---<br>Ligation Product 1.2<br>Linker^{15L}-FN3	--- BtsI + **SpeI** ---<br>Ligation Product 1.3<br>Linker^{7S}-TVMV
3	1	--- BsrDI + **SpeI** + rSAP ---<br>Ligation Product 2.1<br>MBP-CS^{TEV}-StrepTag-II-AI^{TVMV}-Linker^{7S}- ePDZ-B1	--- BtsI + **SpeI** + BbsI-HF ---<br>Ligation Product 2.2<br>Linker^{15L}-FN3-Linker^{7S}-TVMV
4	1	--- BtsI + **SpeI** ---<br>Ligation Product 3.1<br>MBP-CS^{TEV}-StrepTag-II-AI^{TVMV}-Linker^{7S}-ePDZ-B1-Linker^{15L}-FN3-Linker^{7S}-TVMV	--- BtsI + **NheI** + rSAP ---<br>pFLinkC-XE

Table S2. Library Assembly of Rapamycin-Responsive TVMV Switches: AI-FKBP12-FRB-TVMV

Step	Ligation	N-Terminal Fragment(s)	C-Terminal Fragment(s)
1	1	--- BsrDI + **SpeI** + rSAP --- Linker^{7S} G, GG, GGG, GGSG, GPG, GPPPG, GSPAG	--- BtsI + **SpeI** --- FKBP12
	2	--- BsrDI + **SpeI** + rSAP --- Link^{15L} G, GG, GGG, GGSG, GGSGGSG, GGSGGSGGSG, GGSGGSGGSGGSGSG, GSPAG, GGASPAGG, GGASPAAPAPAG, GPG, GPPPG, GPPPPPPPG, GTPTPTPTPTG, GGAEAAAKEAAAKAGG	--- BtsI + **SpeI** --- FRB
	3	--- BsrDI + **SpeI** + rSAP --- Link^{7S} G, GG, GGG, GGSG, GPG, GPPPG, GSPAG	--- BtsI + **SpeI** --- TVMV
2	1	--- BsrDI + **EcoRI** + rSAP --- MBP-CS^{TEV}-StrepTag-II-AI^{TVMV}	--- BtsI + **EcoRI** --- Ligation Product 1.1 Linker^{7S}-FKBP12
	2	--- BsrDI + **SpeI** + rSAP --- Ligation Product 1.2 Linker^{15L}-FRB	--- BtsI + **SpeI** --- Ligation Product 1.3 Linker^{7S}-TVMV
3	1	--- BsrDI + **SpeI** + rSAP --- Ligation Product 2.1 MBP-CS^{TEV}-StrepTag-II-AI^{TVMV}-Linker^{7S}-FRB	--- BtsI + **SpeI** + BbsI-HF --- Ligation Product 2.2 Linker^{15L}-FRB-Linker^{7S}-TVMV
4	1	--- BtsI + **SpeI** --- Ligation Product 3.1 MBP-CS^{TEV}-StrepTag-II-AI^{TVMV}-Linker^{7S}-FKBP12-Linker^{15L}-FRB-Linker^{7S}-TVMV	--- BtsI + **NheI** + rSAP --- pFLinkC-XE

Quantitative Analysis

Summary of equations used to quantify the apparent K_D of rapamycin-responsive TVMV switches (**Eq. 1**) and the K_i of different AI-domain derivatives (**Eq. 2**) by means of non-linear regression. The K_i was determined with K_M = 65 µM and 5 µM fluorescent TVMV substrate.

Equation 1

$$\boldsymbol{Y = V_0 + (V_{Max} - V_0) \times \frac{([TVMV] + [Ligand] + K_D) - \sqrt{([TVMV] + [Ligand] + K_D)^2 - (4 \times [TVMV] \times [Ligand])}}{2 \times [TVMV]}}$$

Equation 2

$$\boldsymbol{Y = V_{Max} \times \frac{[Substrate]}{[Substrate] + K_M \times (1 + \frac{[Inhibitor]}{1 + Ki})}}$$

Amino Acid Sequences of Functional Domains

ePDZ-B1

SPELGFSISGGVGGRGNPFRPDDDGIFVTRVQPEGPASKLLQPGDKIIQANGYSFINIEHGQAVSLLK
TFQNTVELIIVREVGNGAKQEIRVRVEKD

FN3

GVSSVPTNLEVVAATPTSLLISWDAYRELPVSYYRITYGETGGNSPVQEFTVPGSKSTATISGLKPGV
DYTITVYAHYNYHYYSSPISINYR

FRB

ILWHEMWHEGLEEASRLYFGERNVKGMFEVLEPLHAMMERGPQTLKETSFNQAYGRDLMEAQEWCRKY
MKSGNVKDLTQAWDLYYHVFRRI

FKBP12

VQVETISPGDGRTFPKRGQTCVVHYTGMLEDGKKFDSSRDRNKPFKFMLGKQEVIRGWEEGVAQMSVG
QRAKLTISPDYAYGATGHPGIIPPHATLVFDVELLKLE

MBP-CS^{TEV}-StrepTag-II-AI^{TVMV}

KIEEGKLVIWINGDKGYNGLAEVGKKFEKDTGIKVTVEHPDKLEEKFPQVAATGDGPDIIFWAHDRFG
GYAQSGLLAEITPDKAFQDKLYPFTWDAVRYNGKLIAYPIAVEALSLIYNKDLLPNPPKTWEEIPALD
KELKAKGKSALMFNLQEPYFTWPLIAADGGYAFKYENGKYDIKDVGVDNAGAKAGLTFLVDLIKNKHM
NADTDYSIAEAAFNKGETAMTINGPWAWSNIDTSKVNYGVTVLPTFKGQPSKPFVGVLSAGINAASPN
KELAKEFLENYLLTDEGLEAVNKDKPLGAVALKSYEEELVKDPRIAATMENAQKGEIMPNIPQMSAFW
YAVRTAVINAASGRQTVDEALKDAQTNSSS**ENLYFQS**SGWSHPQFEKSGG**REYVRFAP**

- MBP is underlined dotted; cleavage site for TEV protease in **bold**; StrepTag II affinity purification tag underlined; autoinhibitory domain of TVMV protease **bold and underlined**

TVMV Protease

SKALLKGVRDFNPISACVCLLENSSDGHSERLFGIGFGPYIIANQHLFRRNNGELTIKTMHGEFKVKN
STQLQMKPVEGRDIIVIKMAKDFPPFPQKLKFRQPTIKDRVCMVSTNFQQKSVSSLVSESSHIVHKED
TSFWQHWITTKDGQCGSPLVSIIDGNILGIHSLTHTTNGSNYFVEFPEKFVATYLDAADGWCKNWKFN
ADKISWGSFTLVEDAPED

Nucleotide Sequences: pL2

GCGGCCTTTTTACGGTTCCTGGCCTTTTGCTGGCCTTTTGCTCACATGTTCTTTCCTGCGTTATCCCCTGATTCT
GTGGATAACCGTATTACCGCCAATTGGCTCGCCGCAGCCGAACGACCGAGCGCAGCGAGTCACCCGAAGACCATT
TTGGACGCCAGCAGTGGGCATTGCATCCAGCCCCGAGCAAAAGGAGACCCCTGACTAGTCTCCGGGAGCTGCATG
TGTCAGAGGTTTTCACCGTCATCACCGAAACGCGCGACACGAAAGGGCCTCGTGATACGCCTATTTTTATAGGTT
AATGTCATGATAATAATGGTTTCTTAGACGTCAGGTGGCCTTTTCGGGGAAATGTGCGCGGAACCCCTATTTGTT
TATTTTTCTAAATACATTCAAATATGTATCCGCTCATGAATTAATTCTTAGAAAAACTCATCGAGCATCAAATGA
AACTGCAATTTATTCATATCAGGATTATCAATACCATATTTTTGAAAAAGCCGTTTCTGTAATGAAGGAGAAAAC
TCACCGAGGCAGTTCCATAGGATGGCAAGATCCTGGTATCGGTCTGCGATTCCGACTCGTCCAACATCAATACAA
CCTATTAATTTCCCCTCGTCAAAAATAAGGTTATCAAGTGAGAAATCACCATGAGTGACGACTGAATCCGGTGAG
AATGGCAAAAGTTTATGCATTTCTTTCCAGACTTGTTCAACAGGCCAGCCATTACGCTCGTCATCAAAATCACTC
GCATCAACCAAACCGTTATTCATTCGTGATTGCGCCTGAGCCAGACGAAATACGCGGTCGCTGTTAAAAGGACAA
TTACAAACAGGAATCGAATGCAACCGGCGCAGGAACACCGCCAGCGCATCAACAATATTTTCACCTGAATCAGGA
TATTCTTCTAATACCTGGAATGCTGTTTTCCCTGGGATCGCCGTGGTGAGTAACCATGCATCATCAGGAGTACGG
ATAAAATGCTTGATGGTCGGAAGAGGCATAAATTCCGTCAGCCAGTTTAGTCTGACCATCTCATCTGTAACATCA
TTGGCAACGCTACCTTTGCCATGTTTCAGAAACAACTCTGGCGCATCGGGCTTCCCATACAATCGATAGATTGTC
GCACCTGATTGCCCGACATTATCGCGAGCCCATTTATACCCATATAAATCAGCATCCATGTTGGAATTTAATCGC
GGCCTAGAGCAAGACGTTTCCCGTTGAATATGGCTCATAACACCCCTTGTATTACTGTTTATGTAAGCAGACAGT
TTTATTGTGAATTCTCATGACCAAAATCCCTTAACGTGAGTTTTCGTTCCACTGAGCGTCAGACCCCGTAGAAAA
GATCAAAGGATCTTCTTGAGATCCTTTTTTTCTGCGCGTAATCTGCTGCTTGCAAACAAAAAAACCACCGCTACC
AGCGGTGGTTTGTTTGCCGGATCAAGAGCTACCAACTCTTTTTCCGAAGGTAACTGGCTTCAGCAGAGCGCAGAT
ACCAAATACTGTTCTTCTAGTGTAGCCGTAGTTAGGCCACCACTTCAAGAACTCTGTAGCACCGCCTACATACCT
CGCTCTGCTAATCCTGTTACCAGTGGCTGCTGCCAGTGGCGATAAGTCGTGTCTTACCGGGTTGGACTCAAGACG
ATAGTTACCGGATAAGGCGCAGCGGTCGGGCTGAACGGGGGGTTCGTGCACACAGCCCAGCTTGGAGCGAACGAC
CTACACCGAACTGAGATACCTACAGCGTGAGCTATGAGAAAGCGCCACGCTTCCCGAAGGGAGAAAGGCGGACAG
GTATCCGGTAAGCGGCAGGGTCGGAACAGGAGAGCGCACGAGGGAGCTTCCAGGGGGAAACGCCTGGTATCTTTA
TAGTCCTGTCGGGTTTCGCCACCTCTGACTTGAGCGTCGATTTTTGTGATGCTCGTCAGGGGGGCGGAGCCTATG
GAAAAACGCCAGCAAC

Nucleotide Sequence: pFD

GCGGCCTTTTTACGGTTCCTGGCCTTTTGCTGGCCTTTTGCTCACATGTTCTTTCCTGCGTTATCCCCTGATTCT
GTGGATAACCGTATTACCGCCAATTGAGTGAGCTGATACCGCTCGCCGCAGCCGAACGACCGAGCGCAGCGAGTC
ACCCGCGAAATACAAGAAGACAATTTTCCCGCACCAGCAGTGGGGGCATTGCATAAAGCCCCGACAGTAAAAAGA
GACCCTGACTAGTCTCCGGGAGCTGCATGTGTCAGAGGTTTTCACCGTCATCACCGAAACGCGCGACACGAAAGG
GCCTCGTGATACGCCTATTTTTATAGGTTAATGTCATGATAATAATGGTTTCTTAGACGTCAGGTGGCCTTTTCG
GGGAAATGTGCGCGGAACCCCTATTTGTTTATTTTTCTAAATACATTCAAATATGTATCCGCTCATGAATTAATT
CTTAGAAAAACTCATCGAGCATCAAATGAAACTGCAATTTATTCATATCAGGATTATCAATACCATATTTTTGAA
AAAGCCGTTTCTGTAATGAAGGAGAAAACTCACCGAGGCAGTTCCATAGGATGGCAAGATCCTGGTATCGGTCTG
CGATTCCGACTCGTCCAACATCAATACAACCTATTAATTTCCCCTCGTCAAAAATAAGGTTATCAAGTGAGAAAT
CACCATGAGTGACGACTGAATCCGGTGAGAATGGCAAAAGTTTATGCATTTCTTTCCAGACTTGTTCAACAGGCC
AGCCATTACGCTCGTCATCAAAATCACTCGCATCAACCAAACCGTTATTCATTCGTGATTGCGCCTGAGCCAGAC
GAAATACGCGGTCGCTGTTAAAAGGACAATTACAAACAGGAATCGAATGCAACCGGCGCAGGAACACCGCCAGCG
CATCAACAATATTTTCACCTGAATCAGGATATTCTTCTAATACCTGGAATGCTGTTTTCCCTGGGATCGCCGTGG
TGAGTAACCATGCATCATCAGGAGTACGGATAAAATGCTTGATGGTCGGAAGAGGCATAAATTCCGTCAGCCAGT
TTAGTCTGACCATCTCATCTGTAACATCATTGGCAACGCTACCTTTGCCATGTTTCAGAAACAACTCTGGCGCAT
CGGGCTTCCCATACAATCGATAGATTGTCGCACCTGATTGCCCGACATTATCGCGAGCCCATTTATACCCATATA
AATCAGCATCCATGTTGGAATTTAATCGCGGCCTAGAGCAAGACGTTTCCCGTTGAATATGGCTCATAACACCCC
TTGTATTACTGTTTATGTAAGCAGACAGTTTTATTGTGAATTCTCATGACCAAAATCCCTTAACGTGAGTTTTCG
TTCCACTGAGCGTCAGACCCCGTAGAAAAGATCAAAGGATCTTCTTGAGATCCTTTTTTTCTGCGCGTAATCTGC
TGCTTGCAAACAAAAAAACCACCGCTACCAGCGGTGGTTTGTTTGCCGGATCAAGAGCTACCAACTCTTTTTCCG
AAGGTAACTGGCTTCAGCAGAGCGCAGATACCAAATACTGTTCTTCTAGTGTAGCCGTAGTTAGGCCACCACTTC
AAGAACTCTGTAGCACCGCCTACATACCTCGCTCTGCTAATCCTGTTACCAGTGGCTGCTGCCAGTGGCGATAAG
TCGTGTCTTACCGGGTTGGACTCAAGACGATAGTTACCGGATAAGGCGCAGCGGTCGGGCTGAACGGGGGGTTCG
TGCACACAGCCCAGCTTGGAGCGAACGACCTACACCGAACTGAGATACCTACAGCGTGAGCTATGAGAAAGCGCC
ACGCTTCCCGAAGGGAGAAAGGCGGACAGGTATCCGGTAAGCGGCAGGGTCGGAACAGGAGAGCGCACGAGGGAG
CTTCCAGGGGGAAACGCCTGGTATCTTTATAGTCCTGTCGGGTTTCGCCACCTCTGACTTGAGCGTCGATTTTTG
TGATGCTCGTCAGGGGGGCGGAGCCTATGGAAAAACGCCAGCAAC

Nucleotide Sequence: pFLinkC-XE

GCCGGCCACGATGCGTCCGGCGTAGAGGATCGAGATCTCGATCCCGCGAAATTCTAGAGTAATACGACTCACTAT
AGGGAGAAATAATTTTGTTTAACTTTAAAAAAAAAAAGAAGGAGAATAATCTATGGGGCACTGCATGGGGGTGTC
CAAGGGAGAGGAGGATAATATGGCTTCCCTGCCGGCGACCCATGAACTTCATATCTTTGGTTCGATCAACGGAGT
GGACTTTGATATGGTTGGTCAAGGCACGGGAAACCCGAACGATGGGTATGAAGAGTTGAATCTTAAATCGACAAA
AGGGGATCTTCAATTCAGCCCTTGGATTCTGGTCCCGCATATCGGGTATGGCTTCCACCAGTATTTACCATACCC
GGACGGGATGTCTCCATTCCAGGCCGCTATGGTGGATGGCTCTGGTTACCAAGTGCATCGCACCATGCAGTTCGA
GGACGGAGCATCTCTTACTGTTAACTACCGTTATACCTACGAAGGATCACACATTAAGGGAGAAGCTCAGGTTAA
AGGAACGGGATTTCCCGCTGACGGACCCGTAATGACTAATAGCCTGACAGCTGCCGACTGGTGTCGCAGTAAGAA
AACTTATCCTAACGACAAAACGATTATCTCGACGTTCAAATGGTCTTACACGACTGGAAATGGGAAGCGCTACCG
TAGCACGGCCCGCACGACGTACACCTTTGCCAAACCGATGGCGGCTAACTATCTGAAGAATCAACCCATGTATGT
TTTTCGTAAAACAGAATTGAAACATAGTAAGACTGAACTGAACTTCAAGGAGTGGCAAAAAGCATTTACGGACGT
GATGGGGATGGATGAGTTATATAAAGGTGGATCCGGAGGTAGCTGTCTGAGCTACGACACTGAAATCCTGACGGT
AGAATACGGGTTCCTTCCCATCGGTAAGATCGTTGAAGAACGTATCGAGTGTACTGTTTACACTGTGGACAAAAA
CGGGTTCGTCTATACGCAGCCGATTGCACAATGGCATAATCGCGGCGAACAAGAGGTTTTTGAGTATTGCCTGGA
GGACGGCAGTATCATTCGCGCCACTAAAGATCATAAATTTATGACGACCGATGGTCAGATGTTGCCTATTGATGA
AATCTTCGAACGTGGGCTTGACTTGAAACAAGTTGATGGCTTACCAGGCATTGCATAAGCTAGCCGATTCCAGGC
ATCAAATAAAACGAAAGGCTCAGTCGAAAGACTGGGCCTTTCGTTTTATCTGTTGTTTGTCGGTGAACGCTCTCT
ACTAGAGTCACACTGGCTCACCTTCGGGTGGGCCTTTCTGCGTTTATACTCCGGGAGCTGCATGTGTCAGAGGTT
TAACGAAAGGGCCTCGTGATACGCCTATTTTTATAGGTTAATGTCATGATAATAATGGTTTCTTAGACGTCAGGT
GGCAGGCTTACTTTTCGGGGAAATGTGCGCGGAACCCCTATTTGTTTATTTTTCTAAATACATTCAAATATGTAT
CCGCTCATGAGACAATAACCCTGATAAATGCTTCAATAATATTGAAAAAGGAAGAGTATGAGTATTCAACATTTC
CGTGTCGCCCTTATTCCCTTTTTTGCGGCATTTTGCCTTCCTGTTTTTGCTCACCCAGAAACGCTGGTGAAAGTA
AAAGATGCTGAAGATCAGTTGGGTGCACGAGTGGGTTACATCGAACTGGATCTCAACAGCGGTAAGATCCTTGAG
AGTTTTCGCCCCGAAGAACGTTTTCCAATGATGAGCACTTTTAAAGTTCTGCTATGTGGCGCGGTATTATCCCGT
ATTGACGCCGGGCAAGAGCAACTCGGTCGCCGCATACACTATTCTCAGAATGACTTGGTTGAGTACTCACCAGTC
ACAGAAAAGCATCTTACGGATGGCATGACAGTAAGAGAATTATGTAGTGCTGCCATAACCATGAGTGATAACACA
GCGGCCAACTTACTTCTGACAACGATCGGAGGACCGAAGGAGCTAACCGCTTTTTTGCACAACATGGGGGATCAT
GTAACTCGCCTTGATCGTTGGGAACCGGAGCTGAATGAAGCCATACCAAACGACGAGCGTGACACCACGATGCCT
GTAGCGATGGCAACAACGTTGCGCAAACTATTAACTGGCGAACTACTTACTCTAGCTTCCCGGCAACAATTAATA
GACTGGATGGAGGCGGATAAAGTTGCAGGACCACTTCTTCGCTCAGCACTTCCAGCTGGTTGGTTTATTGCTGAT
AAATCTGGAGCCGGTGAGCGTGGATCTCGCGGTATCATAGCAGCACTGGGGCCAGATGGTAAGCCCTCCCGTATC
GTAGTTATCTACACGACGGGGAGTCAGGCAACTATGGATGAACGAAATAGACAGATCGCTGAGATAGGTGCCTCA
CTGATTAAGCATTGGTAACTGTCAGACCAAGTTTACTCATATATACTTTGAATTCAAACTTCATTTTTAATTTAA
AAGGATCTAGGTGAAGATCCTTTTTGATAATCTCATGACCAAAATCCCTTAACGTGAGTTTTCGTTCCACTGAGC
GTCAGACCCCGTAGAAAAGATCAAAGGATCTTCTTGAGATCCTTTTTTTCTGCGCGTAATCTGCTGCTTGCAAAC
AAAAAAACCACCGCTACCAGCGGTGGTTTGTTTGCCGGATCAAGAGCTACCAACTCTTTTTCCGAAGGTAACTGG
CTTCAGCAGAGCGCAGATACCAAATACTGTTCTTCTAGTGTAGCCGTAGTTAGGCCACCACTTCAAGAACTCTGT
AGCACCGCCTACATACCTCGCTCTGCTAATCCTGTTACCAGTGGCTGCTGCCAGTGGCGATAAGTCGTGTCTTAC
CGGGTTGGACTCAAGACGATAGTTACCGGATAAGGCGCAGCGGTCGGGCTGAACGGGGGGTTCGTGCACACAGCC
CAGCTTGGAGCGAACGACCTACACCGAACTGAGATACCTACAGCGTGAGCTATGAGAAAGCGCCACGCTTCCCGA
AGGGAGAAAGGCGGACAGGTATCCGGTAAGCGGCAGGGTCGGAACAGGAGAGCGCACGAGGGAGCTTCCAGGGGG
AAACGCCTGGTATCTTTATAGTCCTGTCGGGTTTCGCCACCTCTGACTTGAGCGTCGATTTTTGTGATGCTCGTC
AGGGGGGCGGAGCCTATGGAAAAACGCCAGCAACGCGGCCTTTTTACGGTTCCTGGCCTTTTGCTGGCCTTTTGC
TCACATGTTCTTTCCTGCGTTATCCCCTGATTCTGTGGATAACCGTATTACCGCCAATTGATGTCGGCGATATAG
GCGCCAGCAACCGCACCTGTGGCGCCGGTGAT

Table S3. Summary Transformation Efficiencies: Assembly of the AI-FKBP12-FRB-TVMV Library

Cloning Step	Part Description	Extrapolated Number of Transformants	Theoretical Diversity	No. Clones to Get All Variants (p = 0.99)
1.1	Linker^{7S}-FKBP12	20.500	7	30
1.2	Linker^{15L}-FRB	30.400	15	67
1.3	Linker^{7S}-TVMV	7.000	7	30
2.1	Linker^{15L}-FRB-Linker^{7S}-TVMV	8.125	105	481
2.2	MBP-CS^{TEV}-StrepTag-II-AI^{TVMV}-Linker^{7S}-FKBP12	12.125	7	30
3	MBP-CS^{TEV}-StrepTag-II-AI^{TVMV}-Linker^{7S}-FKBP12-Linker^{15L}-FRB-Linker^{7S}-TVMV	36.800	735	3382

Table S4. Library screening experiments AI-FKBP12-FRB-TVMV; Equivalent to Tab. 4, but reordered.

Variant	L1	L2	L3	×-Fold Induction; in Lysates	×-Fold Induction; Purified
1-A9	GPG	G(TP)$_4$TG	GPG	> 80	150.6 ± 5.1
3-F6	GPPPG	G(TP)$_4$TG	G	68.3 ± 17	69.7 ± 8.9
2-B3	GPG	GPPPPPPPG	GPG	72.5 ± 13.7	87.1 ± 6.0
2-E7	GPG	GPPPPPPPG	G	41.0 ± 6.6	
1-C9	GPG	GGAEAAAKEAAAKAGG	GSPAG	> 60	36.9 ± 0.7
2-G2	GPPPG	GGAEAAAKEAAAKAGG	GG	16.8 ± 1.9	
2-E3	GSPAG	GGAEAAAKEAAAKAGG	GSPAG	11.9 ± 0.9	
2-G1	GSPAG	GGAEAAAKEAAAKAGG	GPPPG	4.2 ± 0.5	
3-A1	GPPPG	GPPPG	GG	64.4 ± 21.0	18.7 ± 1.0
3-H4	GPG	(GGS)$_4$GSG	GPPPG	21.6 ± 3.4	
1-C11	GPPPG	(GGS)$_4$GSG	GSPAG	3.6 ± 0.3	
1-E8	GSPAG	(GGS)$_4$GSG	G	2.4 ± 0.2	
1-D7	GSPAG	GGASPAAPAPAG	GSPAG	3.2 ± 0.1	
1-D10	GPPPG	GGASPAAPAPAG	GSPAG	3.0 ± 0.2	
3-C9	GPG	GGG	GSPAG	53.9 ± 9.2	
3-F3	GPG	GSPAG	GGG	38.5 ± 7.5	
1-A7	GPPPG	GG	GGSG	5.0 ± 0.4	
1-A8	GGSG	GPG	GGSG	4.1 ± 0.2	
1-D11	GPPPG	GG	GSPAG	3.6 ± 0.5	
3-A3	GSPAG	GGSG	GPPPG	2.8 ± 0.4	
3-D6	GSPAG	GGG	GPPPG	2.6 ± 0.1	
1-D4	GPPPG	GPG	GSPAG	2.6 ± 0.2	

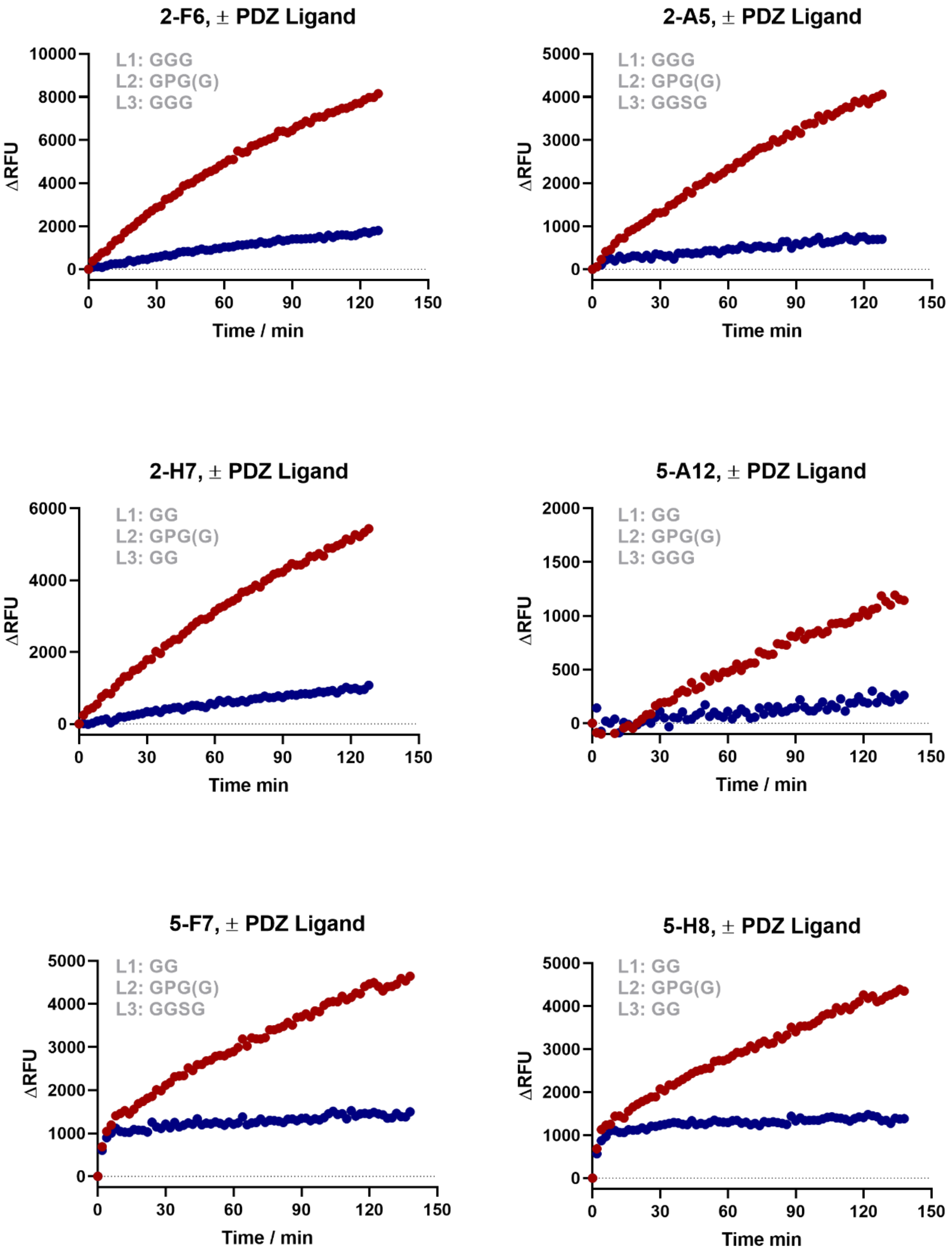

Fig. S1: Kinetic traces of individual mutants sequenced following AI-PDZ-FN3-TVMV library selection experiments. Blue and red traces refer to 10 µM and 0 µM PDZ ligand, respectively. Kinetic traces are measured in cell lysates. Linker sequences and repression values are additionally summarised in Table 3.

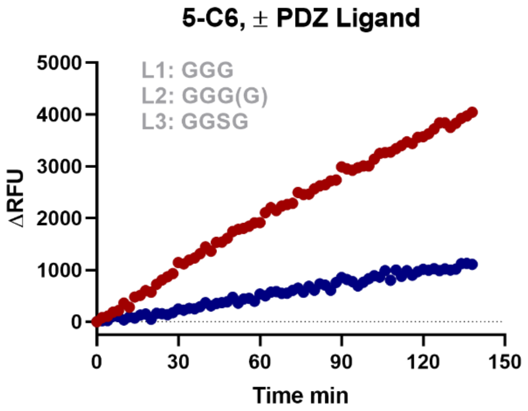

Fig. S2: Kinetic traces of individual mutants sequenced following AI-PDZ-FN3-TVMV library selection experiments. Blue and red traces refer to 10 µM and 0 µM PDZ ligand, respectively. Kinetic traces are measured in cell lysates. Linker sequences and repression values are additionally summarised in Table 3.

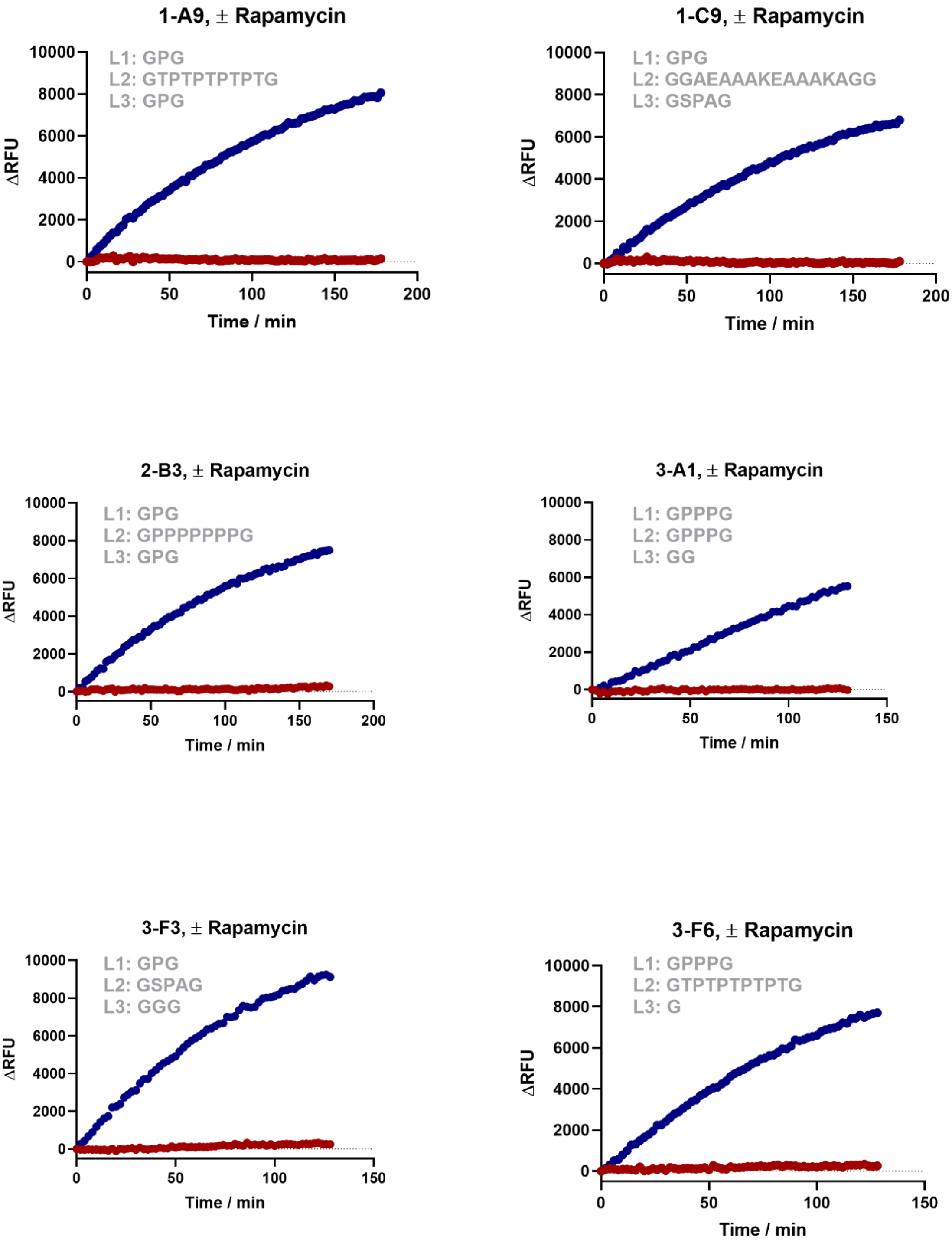

Fig. S3: Kinetic traces of individual mutants sequenced following AI-FKBP12-FRB-TVMV library selection experiments. Blue and red traces refer to 5 µM and 0 µM rapamycin, respectively. Kinetic traces are measured in cell lysates. Linker sequences and induction values are additionally summarised in Table 4.

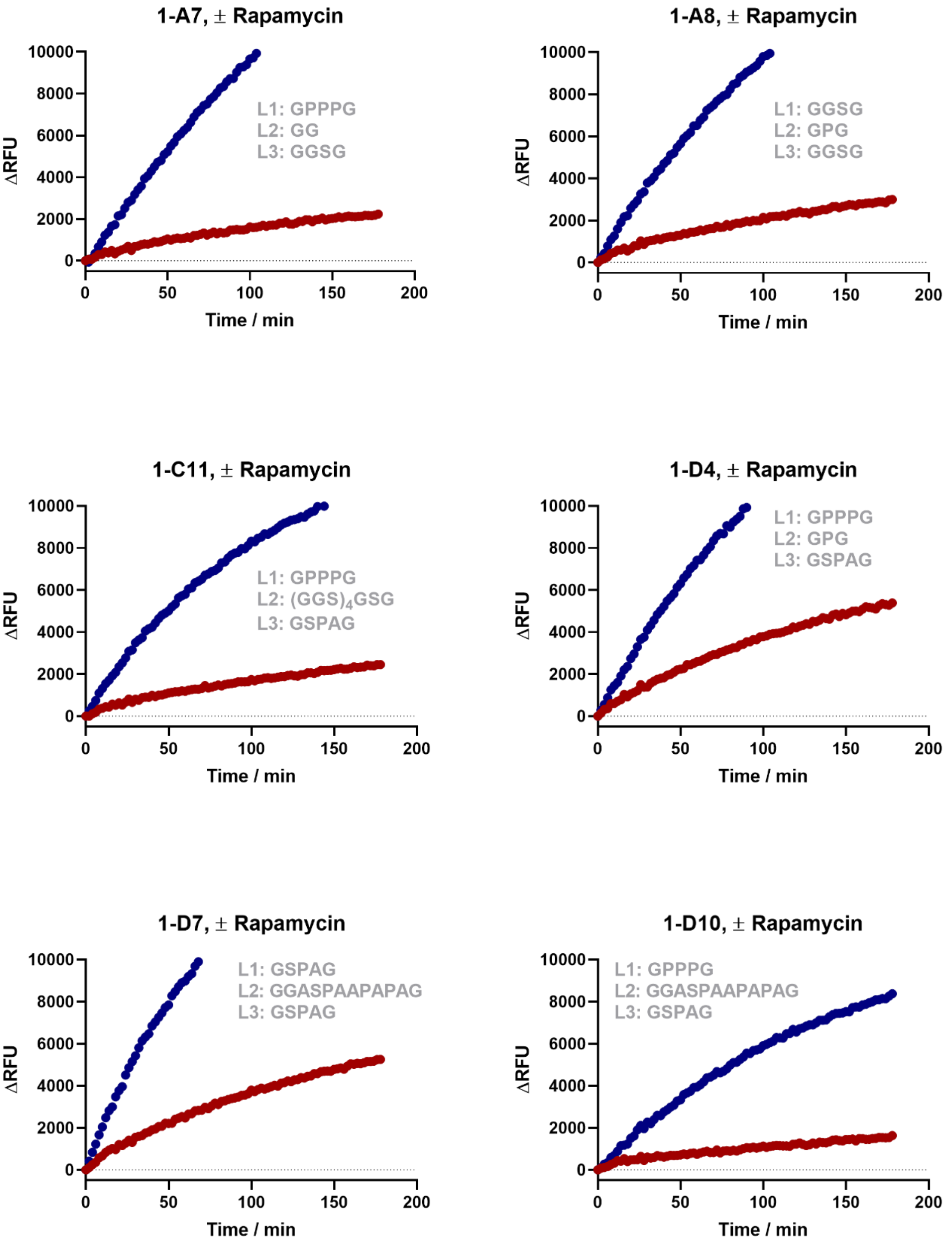

Fig. S4: Kinetic traces of individual mutants sequenced following AI-FKBP12-FRB-TVMV library selection experiments. Blue and red traces refer to 5 μM and 0 μM rapamycin, respectively. Kinetic traces are measured in cell lysates. Linker sequences and induction values are additionally summarised in Table 4.

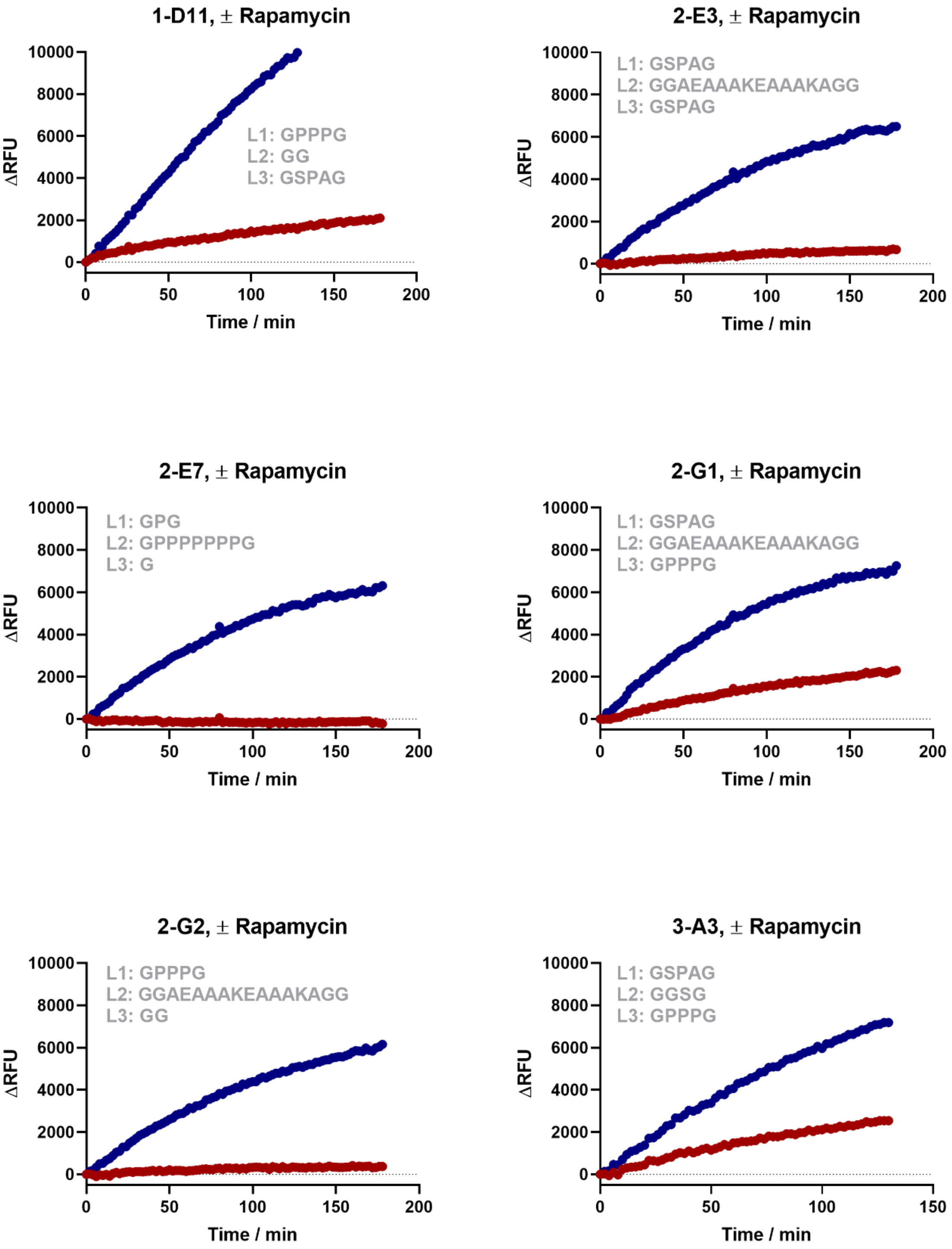

Fig. S5: Kinetic traces of individual mutants sequenced following AI-FKBP12-FRB-TVMV library selection experiments. Blue and red traces refer to 5 μM and 0 μM rapamycin, respectively. Kinetic traces are measured in cell lysates. Linker sequences and induction values are additionally summarised in Table 4.

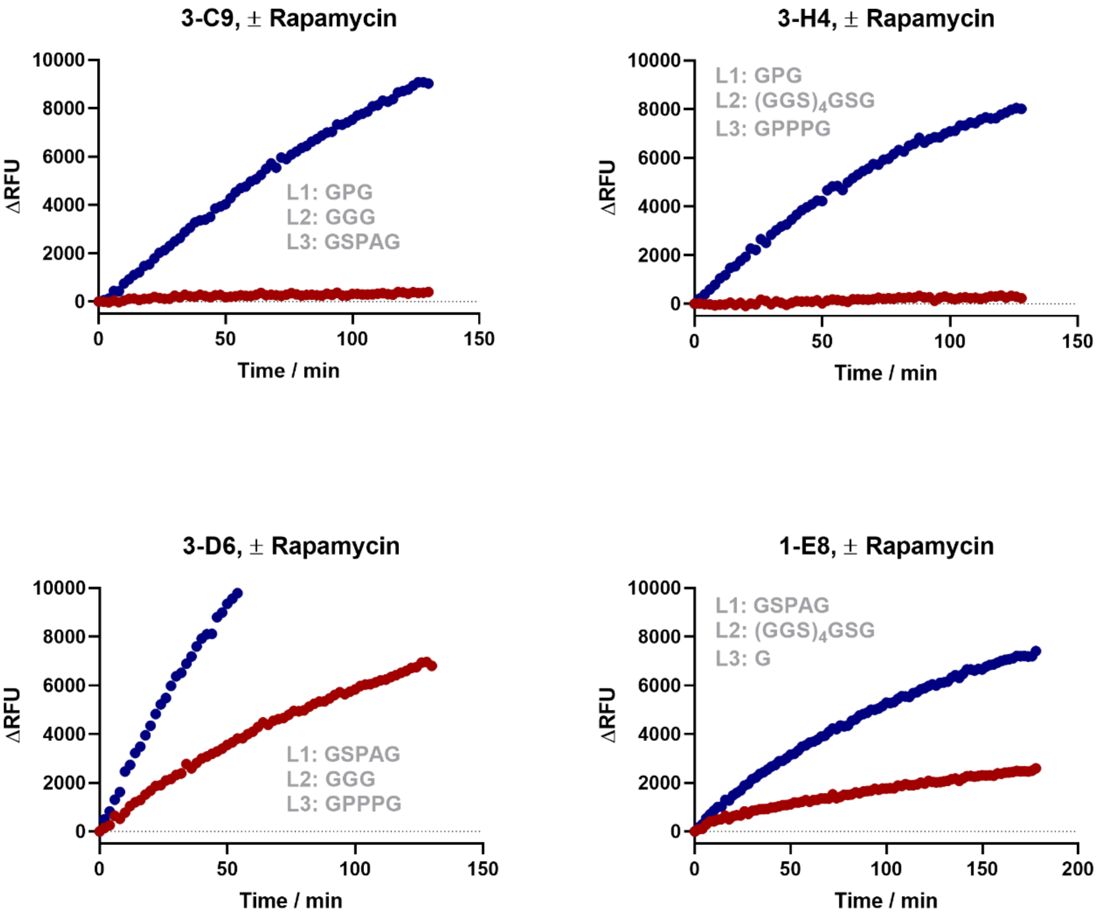

Fig. S6: Kinetic traces of individual mutants sequenced following AI-FKBP12-FRB-TVMV library selection experiments. Blue and red traces refer to 5 µM and 0 µM rapamycin, respectively. Kinetic traces are measured in cell lysates. Linker sequences and induction values are additionally summarised in Table 4.

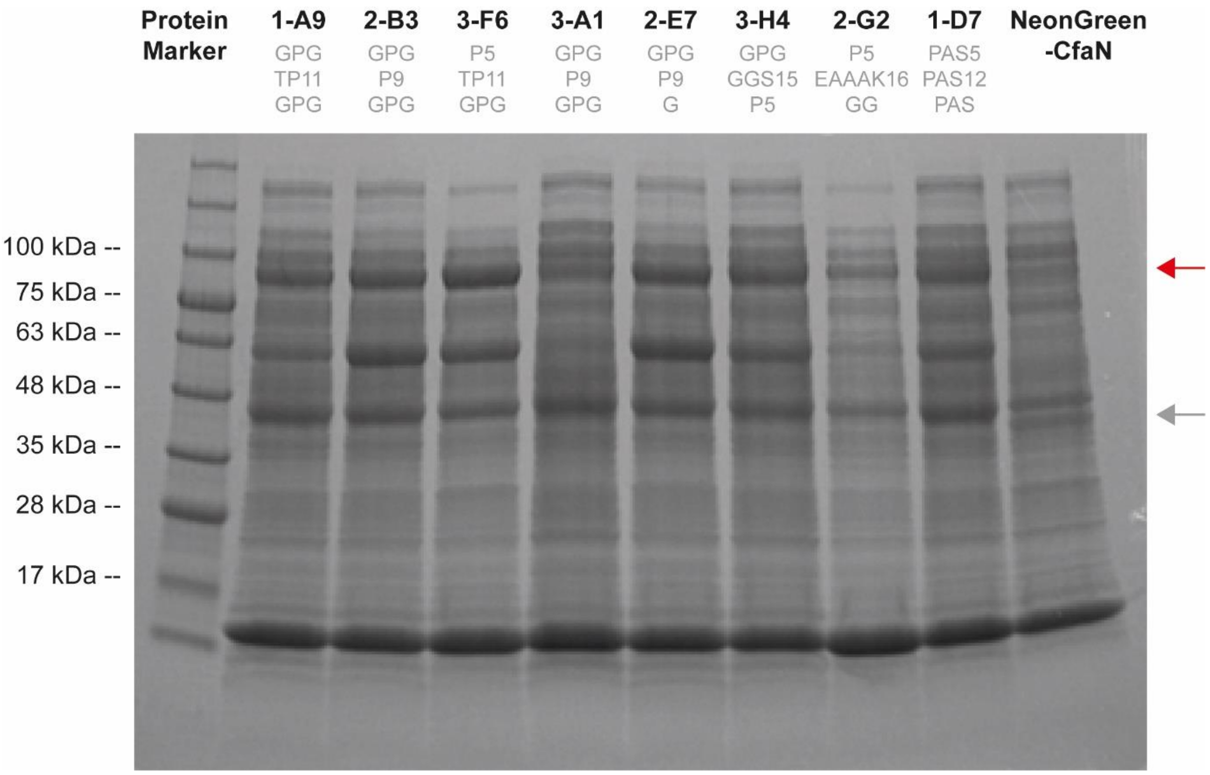

Fig. S7: Protein expression analysis of a select number of AI-FKBP12-FRB-TVMV protease switches. Expression tests were conducted in LB while protein expression was induced with 1 mM IPTG during exponential growth phase and left to express for 3 h. Aliquots of the cell suspension were denatured for 10 min at 95 ˚C in SDS-PAGE loading buffer. Expression analysis shows AI-FKBP12-FRB-TVMV express well with an approx. molecular weight of >90 kDa for the full length MBP-CS^{TEV}-StrepTag-II-AI-FKBP12-FRB-TVMV fusion protein (see red arrow). Two additional bands are visible at approx. 40 kDa and 52 kDa corresponding to non-specific cleavage products of the MBP and AI-FKBP12-FRB-TVMV protease switch. iFLinkC-XE with an mNeonGreen-CfaN insert of approx. 35 kDa (see grey arrow) served as the negative control.
